# LDH and hemoglobin outperform systemic inflammatory indices as prognostic factors in patients with soft tissue sarcoma undergoing neoadjuvant treatment

**DOI:** 10.1186/s12885-025-13889-4

**Published:** 2025-03-18

**Authors:** Luc M. Berclaz, Dorit Di Gioia, Vindi Jurinovic, Michael Völkl, Sinan E. Güler, Markus Albertsmeier, Alexander Klein, Hans Roland Dürr, Sina Mansoorian, Thomas Knösel, Wolfgang G. Kunz, Michael von Bergwelt-Baildon, Lars H. Lindner, Anton Burkhard-Meier

**Affiliations:** 1 Department of Internal Medicine III, University Hospital, LMU Munich, 81377 Munich, Germany; 2 German Cancer Consortium (DKTK), Partner Site Munich, Munich, Germany; 3 Institute for Medical Information Processing, Biometry, and Epidemiology, University Hospital, LMU Munich, Munich, Germany; 4 Department of General, Visceral and Transplantation Surgery, University Hospital, LMU Munich, Munich, Germany; 5 Orthopaedic Oncology, Department of Orthopaedics and Trauma Surgery, University Hospital, LMU Munich, Munich, Germany; 6 Department of Radiation Oncology, University Hospital, LMU Munich, Munich, Germany; 7 Institute of Pathology, LMU Munich, Munich, Germany; 8 Department of Radiology, University Hospital, LMU Munich, Munich, Germany

**Keywords:** Systemic inflammatory index, Soft tissue sarcoma, Chemotherapy, Regional hyperthermia, LDH, Hemoglobin

## Abstract

**Background:**

The current understanding of the prognostic value of routine pre-treatment laboratory parameters in patients with high-risk soft tissue sarcoma (HR-STS) is limited. We sought to analyze several inflammatory biomarkers in a large cohort of HR-STS patients undergoing neoadjuvant therapy followed by curative surgical resection.

**Methods:**

123 patients with locally advanced high-risk undifferentiated pleomorphic sarcoma (UPS), liposarcoma (LPS), leiomyosarcoma (LMS), and synovial sarcoma (SS) who underwent preoperative chemotherapy and regional hyperthermia (RHT) between 2014 and 2022 were retrospectively evaluated. The association of several pre-treatment laboratory parameters with radiologic treatment response, event-free survival (EFS), and overall survival (OS), were analyzed.

**Results:**

Low pre-treatment hemoglobin (HR 2.51, *p* = 0.018; HR 2.78, *p* = 0.030) and lactate dehydrogenase (LDH, HR 0.29, *p* = 0.0044; HR 0.23, *p* = 0.010) were significantly associated with EFS and OS in the multivariable analysis. Systemic inflammatory indices such as the neutrophil-to-lymphocyte ratio (NLR) did not have a significant impact on survival. Low C-reactive protein (CRP) and high albumin values were associated with poor radiologic response according to RECIST (*p* = 0.021 and *p* = 0.010, respectively).

**Conclusion:**

Pre-treatment LDH and hemoglobin are strong independent predictors of survival in HR-STS patients. Systemic inflammatory indices based on circulating immune cells may not serve as reliable prognostic factors for HR-STS patients undergoing curative-intent treatment. Higher pre-treatment albumin levels and lower CRP values may reflect a reduced inflammatory status and could be associated with a poorer radiologic response to preoperative treatment.

**Supplementary information:**

The online version contains supplementary material available at 10.1186/s12885-025-13889-4.

## Introduction

Soft tissue sarcomas (STS) are rare tumors with multiple distinct histological subtypes. They account for approximately 1% of adult malignancies [1]. Despite optimal local treatment, almost half of patients with high risk features (HR-STS: Tumor diameter 5 cm or larger, grade 2 or 3 according to Fédération Nationale des Centres de Lutte Contre le Cancer (FNCLCC), deep to the fascia) will die within five years of their diagnosis [2, 3]. Perioperative chemotherapy is often recommended in addition to surgery and radiotherapy in patients with HR-STS [4, 5]. The addition of regional hyperthermia (RHT) to neoadjuvant chemotherapy has shown to improve both response and survival in HR-STS and is therefore being considered as an additional treatment option according to the ESMO-EURACAN-GENTURIS guidelines [6, 7]. This is mostly due to a large multicenter phase III trial that demonstrated a 10-year overall survival (OS) benefit of 9.9% with chemotherapy and RHT compared to chemotherapy alone [6, 7]. Moreover, preoperative radiotherapy is widely accepted as standard treatment for patients with extremity sarcomas undergoing limb-sparing surgery [8]. For retroperitoneal sarcomas (RPS), it is currently limited to certain subtypes [9].

Prognostic factors associated with response to systemic treatment and survival are currently limited and mostly based on basic clinical parameters such as patient age, grade, completeness of surgical resection and histology [2, 10, 11]. Serum inflammatory markers and indices based on circulating immune cells and other laboratory values such as C-reactive protein (CRP) or lactate dehydrogenase (LDH) have been proposed as additional tools to predict response to treatment and clinical outcomes in various cancer types [12–14]. In STS, differences in these biomarkers across tumor sites, histological subtypes and extent of disease are not fully understood [15, 16]. Fiore et al. were able to correlate an increased neutrophil-to-lymphocyte ratio (NLR) and composite inflammatory biomarkers prognostic index (IBPI) with worse OS in patients with primary resectable RPS [17]. In contrast, Choi et al. observed that CRP and erythrocyte sedimentation rate remained the only predictive laboratory values of survival in localized STS [18]. In addition to survival, Fausti et al. linked a high lymphocyte-to-monocyte ratio (LMR) with better response to second-line chemotherapy with trabectedin in metastatic STS patients [19], which underlines the predictive potential of routine laboratory values in daily clinical practice.

Aim of this study was to correlate inflammation-related laboratory parameters with clinical characteristics, radiologic treatment response and survival in HR-STS patients undergoing a multimodal neoadjuvant treatment approach including chemotherapy, RHT, and radiotherapy in selected cases.

## Materials and methods

### Patient selection

An exploratory retrospective cohort study design was chosen to address the research question. Eligible adult (≥ 18 years) patients had pathologically confirmed locally advanced high-risk undifferentiated pleomorphic sarcoma (UPS), liposarcoma (LPS), leiomyosarcoma (LMS), or synovial sarcoma (SS) without evidence of metastasis and were treated at our institution between January 2014 and May 2022. Clinical, pathologic, and outcomes data were extracted from our prospectively maintained clinical sarcoma database. Patients received up to eight cycles of doxorubicin in combination with ifosfamide (AI) or doxorubicin in combination with dacarbazine (AD) in patients with LMS treated after January 2020 due to a change in in-house protocol for this histological subtype. Patients < 60 years of age received 60mg/m^2^ of doxorubicin per cycle and 9 g/m^2^ of ifosfamide (decreased to 6 g/m^2^ for cycles 5–8) or 1200mg/m^2^ of dacarbazine. The standard dose for patients ≥ 60 years was 60mg/m^2^ of doxorubicin combined with either 6 g/m^2^ of ifosfamide or 900mg/m^2^ of dacarbazine per cycle. All patients were treated with chemotherapy in combination with RHT. RHT aiming for tumor temperatures elevating to 40°-43 °C for 60 min was given twice per chemotherapy cycle. Quality and safety of hyperthermia was ensured by the European Society for Hyperthermic Oncology (ESHO) guidelines [20]. The BSD-2000 hyperthermia system (PYREXAR Medical, Salt Lake City, UT, USA) was used. Surgery was generally performed after four cycles of chemotherapy and RHT. In case of tumor progression detected on CT imaging after two cycles, chemotherapy was discontinued, and surgery was performed earlier. Radiotherapy was used in a pre- or postoperative setting in patients with extremity sarcomas or in selected non-extremity cases to enhance local tumor control after discussion in our multidisciplinary sarcoma tumor board. Patients with evidence of metastasis prior to the start of neoadjuvant treatment, prior chemotherapy, medical conditions known to affect the inflammatory parameters and white blood cell counts (such as hematologic disorders, active infection, or history of glucocorticoid use), or no resection were excluded from this analysis.

### Systemic inflammatory parameters

The following laboratory parameters were reviewed: hemoglobin, platelets, CRP, LDH, albumin, leukocytes, neutrophils, lymphocytes, monocytes, neutrophil-to-lymphocyte ratio (NLR), lymphocyte-to-monocyte ratio (LMR), platelet-to-lymphocyte ratio (PLR), CRP-to-lymphocyte ratio (CLR). The laboratory parameters were collected within seven days prior to the beginning of neoadjuvant chemotherapy and RHT.

### Radiologic assessments

Radiologic tumor response after two cycles of chemotherapy and RHT was assessed in all patients according to the response evaluation criteria in solid tumors (RECIST) 1.1 [21]. Imaging was reviewed by a radiologist with subspecialty training in oncologic imaging and extensive experience in sarcoma imaging (WGK).

### Statistical analysis

Survival endpoints of this study included event-free survival (EFS) and OS. The EFS duration was estimated by the time from start of chemotherapy and RHT to first progression, recurrence, or death. OS was estimated by the time from start of chemotherapy to death by any cause. Cox proportional hazards regression analyses were performed to assess the association between various clinical variables and EFS/OS, with event-free patients being censored at the time of their last follow-up visit. Logistic regression analyses were conducted to evaluate the association between clinical variables and radiologic treatment response. Pearson’s correlation coefficients were calculated to assess the relationship between different laboratory parameters. Laboratory parameters were dichotomized using a median split. Laboratory parameters were compared across histological subtypes using the Kruskal-Wallis test, followed by Dunn’s post-hoc test with Bonferroni correction for multiple comparisons. A p-value ≤ 0.05 was considered statistically significant. Statistical analyses were performed using R version 4.3.3 (R Foundation for Statistical Computing, Vienna, Austria).

## Results

### Patient cohort

A total of 123 patients were analyzed. The clinicopathologic characteristics of the study cohort are summarized in Table 1. Chemotherapy data can be seen in Table 2. Median age was 62 years (range 25–89 years). The most common histological subtype was UPS (35%), followed by LMS (28%) and LPS (25%). Almost all patients had extremity (48%) or intraabdominal/retroperitoneal (41%) STS. The median tumor size was 10 cm (range 3–52 cm). 76% of patients had stable disease (SD) according to RECIST after two cycles of neoadjuvant chemotherapy and RHT.

**Table 1:** Patient characteristics

Variable	Strata	*n* (%)
Age	Median [range]	62 [25–79]
Sex	Female	57 (46)
	Male	66 (54)
Recurrent tumor	Yes	10 (8)
	No	113 (92)
Largest diameter of primary tumor (cm)	Median [range]	10 [3–52]
Histology	UPS	43 (35)
	LMS	35 (28)
	LPS • Dedifferentiated LPS • Pleomorphic LPS • Myxoid LPS	31 (25) • 23 (19) • 3 (2) • 5 (4)
	SS	14 (11)
Grade	2	57 (46)
	3	66 (54)
Site of primary tumor	Extremity	59 (48)
	Intraabdominal/ retroperitoneal	50 (41)
	Other	14 (11)
Surgery	Yes	123 (100)
	No	0 (0)
Extent of surgery	R0	107 (87)
	R1	13 (11)
	RX	3 (2)
Radiotherapy	Yes	88 (72)
	No	35 (28)
Radiologic Response (RECIST)	CR PR SD PD	0 (0) 9 (7) 94 (76) 20 (16)

UPS = Undifferentiated pleomorphic sarcoma, LMS = Leiomyosarcoma, LPS = Liposarcoma, SS = Synovial sarcoma, CR = Complete response, PR = Partial response, SD = Stable disease, PD = Progressive disease, RECIST: Response Evaluation Criteria in Solid Tumors

**Table 2:** Chemotherapy data

Variable	Strata	*n* (%)
Total chemotherapy cycles		Median [range] 5 [1–8]
Chemotherapy protocol	AI	107 (87)
	AD	16 (13)
Neoadjuvant chemotherapy	Yes	123 (100)
	No	0 (0)
Neoadjuvant chemotherapy cycles	Median [range]	4 [1–8]
Dose reduction	Yes	26 (21)
	No	97 (79)
Reason for dose reduction	Hematological toxicity	25 (96)
	Other	1 (4)

AI = Doxorubicin + Ifosfamide, AD = Doxorubicin + Dacarbazine

### Correlation of systemic inflammatory markers with clinical parameters and radiologic response criteria

The median values and ranges of all laboratory parameters are available as a supplementary file (Supp. Table 1). Common laboratory parameters such as leukocytes, hemoglobin, platelets, and CRP were available in 100% of patients (*n* = 123). LDH was available in 98% of patients (*n* = 121). Neutrophil counts were available in 88% of patients (*n* = 108). Albumin values were available in 86% of patients (*n* = 106). Lymphocyte and monocyte counts were available in 81% of patients (*n* = 99). A comparison of the laboratory parameters across the different histological subtypes included in this study is also provided as a supplementary file (Supp. Tables 2 and 3). Significant differences were observed in several parameters: Leukocytes and neutrophils were higher in UPS than in SS (adj. *p* = 0.018 and *p* = 0.0011, respectively). Additionally, neutrophils were elevated in LPS compared to SS (*p* = 0.028). Hemoglobin was increased in SS compared to LPS and UPS (adj. *p* = 0.018 and *p* = 0.027, respectively). Platelets were higher in UPS than in LMS and SS (adj. *p* = 0.0075 and *p* = 0.0050, respectively). LDH was higher in LMS and UPS than in LPS (adj. *p* < 0.001 and *p* = 0.016, respectively), while Albumin was lower in UPS and LPS compared to SS (adj. *p* = 0.0013 and *p* = 0.030, respectively). CRP was increased in UPS compared to SS (*p* = 0.012). Albumin values were negatively correlated with NLR (r -0.34, *p* = 0.001), PLR (r -0.51, *p* < 0.001), CLR (r -0.60, *p* < 0.001), CRP (r -0.67, *p* < 0.001), leukocytes (r -0.31, *p* = 0.0012), monocytes (r -0.31, *p* = 0.003) and platelets (r -0.57, *p* < 0.001). Albumin was positively correlated with hemoglobin (r 0.67, *p* < 0.001). In logistic regression analysis, high albumin (*p* = 0.010) and low CRP values (*p* = 0.021) were the only laboratory inflammatory parameters associated with poor radiologic response according to RECIST (Table 3). A trend towards poor radiologic response was visible in patients with low PLR (*p* = 0.052).

**Table 3:** Analysis of systemic inflammatory indices on radiologic treatment response

		Radiologic Response (RECIST)		Sig.
Factor	Strata	PR/SD	PD	
LMR	< 2.3	46	4	0.14
	≥ 2.3	40	9	
NLR	< 3.7	41	9	0.16
	≥ 3.7	45	4	
CLR	< 0.62	42	8	0.40
	≥ 0.62	44	5	
PLR	< 212.7	40	10	0.052
	≥ 212.7	46	3	
Albumin (mg/dl)	<4.1	53	4	**0.010**
	≥ 4.1	36	13	
CRP (mg/dl)	< 1.0	47	15	**0.021**
	≥ 1.0	56	5	
LDH (U/l)	< 213	53	8	0.31
	≥ 213	48	12	
Leukocytes (G/l)	< 7.6	50	12	0.35
	≥ 7.6	53	8	
Neutrophils (G/l)	< 5.3	44	10	0.43
	≥ 5.3	47	7	
Lymphocytes (G/l)	< 1.5	44	6	0.74
	≥ 1.5	42	7	
Monocytes (G/l)	< 0.6	42	8	0.40
	≥ 0.6	44	5	
Platelets (G/l)	< 296	50	12	0.35
	≥ 296	53	8	
Hemoglobin (g/dl)	< 13.0	56	8	0.24
	≥ 13.0	47	12	

NLR = Neutrophil-to-lymphocyte ratio, CLR = CRP-to-lymphocyte ratio, PLR = Platelet-to-lymphocyte ratio, CRP = C-reactive protein, LDH = Lactate dehydrogenase, RECIST = Response Evaluation Criteria In Solid Tumors

### Association of patient characteristics and systemic inflammatory indices with survival

**Fig. 1:**
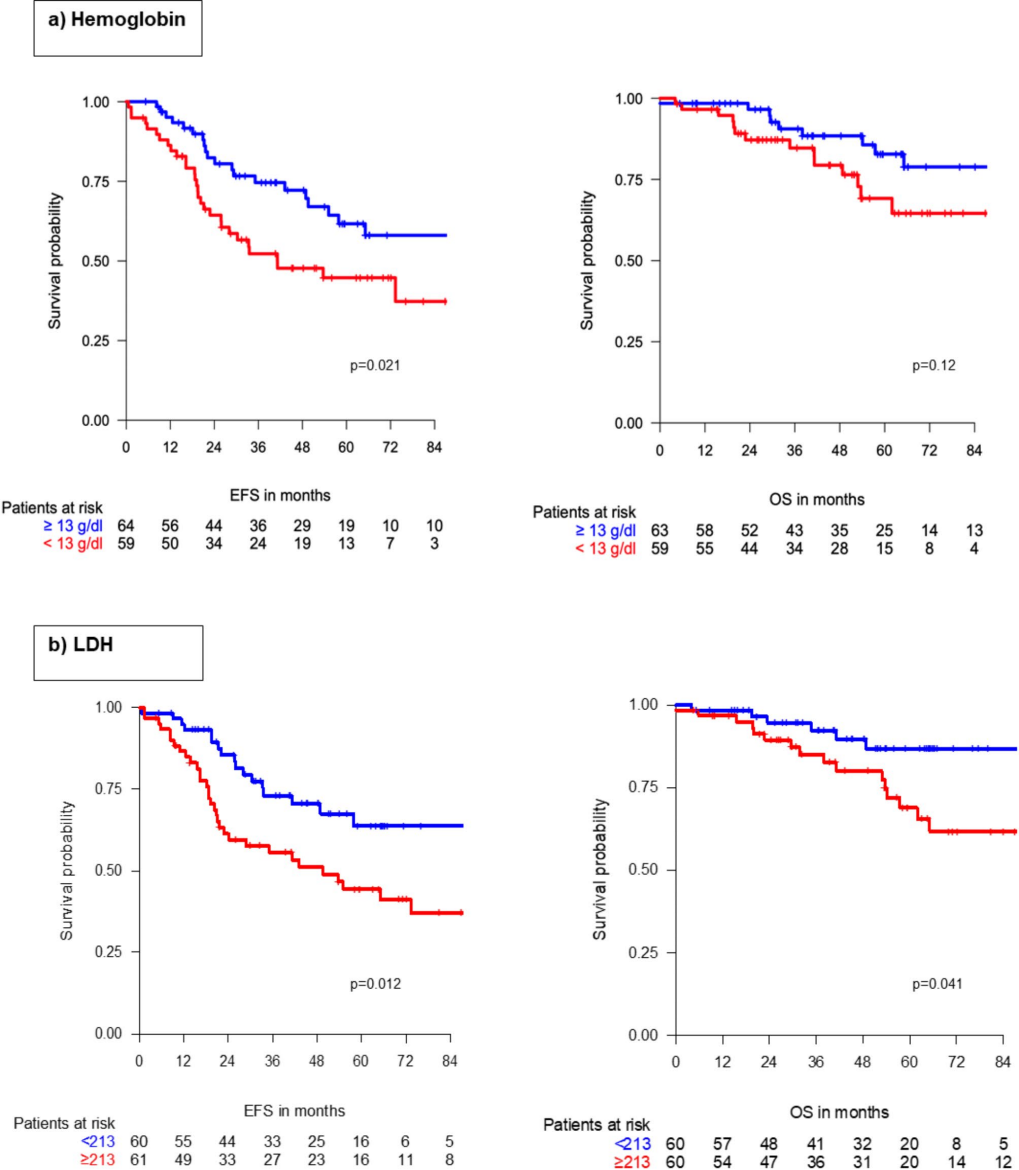
Event-free survival (EFS) and overall survival (OS) according to hemoglobin (**a**) and lactate dehydrogenase (LDH; **b**) levels

The median EFS was 73.3 months (95% CI 48.9– NR), and the median OS was not reached at a median follow-up of 59.6 months (95% CI 51.8–66.2 months). 50 EFS events (41%) and 24 deaths (20%) were reported by the end of follow-up. Univariate analysis of relevant patient characteristics on EFS and OS is available as a supplementary file (Supp. Table 4). Resection margins (HR 0.44, 95% CI 0.22–0.86, *p* = 0.017) and radiotherapy (HR 0.47, 95% CI 0.26–0.83, *p* = 0.010) were significant predictors of EFS. Recurrent disease was associated with an improved EFS in the univariate analysis (HR 2.59, *p* = 0.029). Radiologic disease stabilization or response (PR/SD according to RECIST) after two cycles of neoadjuvant chemotherapy and RHT was significantly associated with a better OS (HR 0.30, 95% CI 0.13–0.71, *p* = 0.0063). Regarding routine laboratory parameters, both hemoglobin < 13 g/dl (HR 1.94, *p* = 0.021) and LDH < 213 U/l (HR 0.47, *p* = 0.012) were the only inflammatory markers associated with EFS. LDH < 213 U/l served as the only laboratory parameter significantly associated with improved OS in the univariate analysis (HR 0.38, *p* = 0.041) (Supp. Table 5) (Fig. 1).

In the multivariable analysis, low pre-treatment LDH and hemoglobin levels remained independent predictors of EFS (LDH: HR 0.29, *p* = 0.0044; Hemoglobin: HR 2.51, *p* = 0.018) and OS (LDH: HR 0.23, *p* = 0.010; Hemoglobin: HR 2.78, *p* = 0.030), respectively. Negative resection margins (EFS: HR 0.34, *p* = 0.025) and radiologic response to chemotherapy and RHT (OS: HR 0.27, *p* = 0.0068) were also significantly associated with improved survival in the multivariable analysis (Table 4).

Exploring various cut-off values for LDH to predict EFS and OS, the lowest p-value was observed at 223 U/l (EFS HR 2.62, *p* = 0.0011; OS HR 3.80, *p* = 0.0048; refence ≥ 223 U/l). For hemoglobin, the optimal cut-off value for both EFS and OS was 12.9 g/dl (EFS HR 1.94, *p* = 0.021; OS HR 1.91, *p* = 0.12; reference: ≥12.9 g/dl).

**Table 4:** Multivariable analysis of relevant clinical and laboratory parameters on event-free and overall survival

		EFS		OS	
Factor	Strata	Sig.	Hazard Ratio (95%CI)	Sig.	Hazard Ratio (95%CI)
Age (years)	< 62 vs. ≥62	0.53	1.26 (0.61–2.60)	0.58	1.32 (0.49–3.57)
Disease status	Primary vs. recurrent	0.66	1.33 (0.38–4.72)	-	-
Radiologic response (RECIST)	PR/SD vs. PD	0.19	0.54 (0.22–1.36)	**0.0068**	**0.27 (0.10–0.70)**
Resection margins	R0 vs. R1-RX	**0.025**	**0.34 (0.14–0.88)**	-	-
Albumin (mg/dl)	< 4.1 vs. ≥4.1	0.51	0.76 (0.34–1.71)	-	-
LDH (U/l)	< 213 vs. ≥213	**0.0044**	**0.29 (0.13–0.68)**	**0.010**	**0.23 (0.07–0.70)**
NLR	< 3.7 vs. ≥3.7	0.88	0.95 (0.46–1.96)	0.15	0.46 (0.16–1.34)
Hemoglobin (g/dl)	< 13.0 Vs. ≥13.0	**0.018**	**2.51 (1.17–5.38)**	**0.030**	**2.78 (1.11–6.97)**

LDH = Lactate dehydrogenase, NLR = Neutrophil-to-lymphocyte ratio, PR = partial response, SD = Stable disease, PD = Progressive disease, RECIST = Response Evaluation Criteria in Solid Tumors

## Discussion

In this series of 123 patients with the most common HR-STS subtypes undergoing neoadjuvant treatment followed by surgery, we found a significant association between pre-treatment hemoglobin and LDH levels and survival. Circulating immune cells and inflammatory indices such as NLR and PLR did not have a significant impact on survival. Furthermore, this is the first study to examine the impact of various inflammatory blood parameters on radiologic treatment response in HR-STS. Our findings suggest that a lower inflammatory status, characterized by higher albumin and lower CRP levels, is associated with a poorer treatment response. Moreover, we observe significant differences in inflammatory parameters among the most common histological subtypes.

In contrast to chemotherapy-induced or postoperative anemia, the cause of cancer-related anemia is multifactorial and often linked to the production of inflammatory cytokines. Previous studies have demonstrated the negative prognostic impact of low preoperative hemoglobin levels in patients with a broad range of STS subtypes [22–24]. Fiore et al. also linked pre-treatment anemia with worse outcomes in patients with retroperitoneal sarcoma and used low hemoglobin levels as a part of an inflammatory biomarkers prognostic index (IBPI) [17].

High LDH levels have also been associated with poor survival in various solid and hematological malignancies and are included in several prognostic nomograms for hematological malignancies like B-cell lymphomas [25–27]. In contrast, only few studies have analyzed the impact of serum LDH levels on survival in STS. In a retrospective study by Lin et al., high preoperative serum LDH was an independent predictor of poor OS and shorter time to recurrence in patients with UPS [28]. Fujibuchi et al. demonstrated a higher risk of metastasis and shorter disease-specific survival in bone and soft tissue sarcomas [13]. In contrast, elevated pre-treatment serum LDH levels were not predictive of survival in adult STS patients in a study by Nakamura et al. [29]. In addition to its prognostic value, Zoghbi et al. demonstrated the potential of serum LDH (combined with NLR in a lung immune prognostic index / LIPI) as a readily available tool for therapy monitoring in STS patients treated with immunotherapy in early-phase trials [30]. In our study, LDH emerged as the most reliable prognostic factor. The serum LDH value of 223 U/l was identified as the optimal cut-off in this analysis. Interestingly, this value is below the upper limit of normal (ULN) for the reference range. These findings underscore the importance of carefully reconsidering “normal” baseline LDH levels in patients with HR-STS.

In our study, low CRP and high albumin values were significantly associated with poor radiologic treatment response. While high albumin and low CRP values were generally favorable prognostic factors in previous studies and associated with better treatment response [31, 32], our data could indicate a lack of systemic immune activation necessary for treatment response to RHT and chemotherapy. Due to the limited number of patients with PR and PD, a multivariable analysis on treatment response could not be conducted. Thus, our findings are hypothesis-generating and should be interpreted with caution. Moreover, the analysis of follow-up CRP values during neoadjuvant treatment would be of interest to detect potential CRP flares, as early dynamic CRP changes have been associated with favorable treatment response in other cancers [33].

Inflammatory indices such as the NLR and PLR have been proposed as an additional prognostic tool in STS, with different results so far. In a large monocentric analysis by Fiore et al., a high NLR and PLR were both significantly associated with worse OS as single biomarkers and as part of the IBPI [17]. In smaller retrospective studies by Szkandera et al. and Viñal et al., the NLR was confirmed as an independent prognostic biomarker in both localized and metastatic STS patients [34, 35]. In contrast, Schwartz et al. demonstrated no prognostic value of both NLR and PLR compared to basic clinical parameters regarding recurrence-free survival (RFS) and OS in 409 patients with mostly localized retroperitoneal and truncal STS [36]. They performed a thorough workup of potential inflammatory comorbid conditions such as tobacco abuse, sepsis or coronary artery disease and applied different cut-offs for both NLR and PLR. Interestingly, when inflammatory comorbidities were excluded from the multivariable analysis, PLR became an independent predictor of RFS, which demonstrates the potential bias in other retrospective studies on this subject. The currently inconclusive findings on inflammatory indices based on routine laboratory values, including our results, highlight the need for prospective studies and registries, such as the global observational study of RPS (RESAR, NCT03838718).

The collected laboratory parameters were compared within the different histological subtypes. We found that most systemic inflammatory markers were elevated in UPS compared to the other histological subtypes. This is in line with previous literature, where UPS is recognized as an inflammatory subtype with a high immune cell infiltration [37–39].

In addition to its retrospective nature, a potential limitation of this study is a possible selection bias due to the chosen treatment regimen: neoadjuvant combination chemotherapy with RHT is usually reserved for patients with good performance status, while patients with lower performance status often undergo direct tumor resection or doxorubicin monotherapy without RHT in advanced cases. As the median OS was not reached in this study on HR-STS patients undergoing curative-intent neoadjuvant therapy and tumor resection, the preliminary results of this study are hypothesis-generating, and optimal risk stratification in these patients should still include known prognostic parameters such as tumor site and size, tumor grading and histological subtype. Based on the results of this large single-center analysis, we believe that patients with high pre-treatment LDH levels and cancer-related anemia should receive their treatment without delay. A subsequent analysis with a longer follow-up and higher case numbers should be conducted in the future, and the impact of follow-up laboratory values during neoadjuvant treatment could bring more insights into the predictive and prognostic role of routine laboratory inflammatory markers. Moreover, a standardized cut-off for inflammatory indices in STS patients would greatly aid in the comparison of future studies.

## Conclusion

Pre-treatment LDH and hemoglobin are strong independent predictors of survival in HR-STS patients. However, systemic inflammatory indices based on circulating immune cells may not serve as reliable prognostic factors for HR-STS patients undergoing curative-intent treatment. Higher pre-treatment albumin levels and lower CRP values may reflect a reduced inflammatory status and could be associated with a poorer radiologic response to preoperative treatment. These findings should be validated in a larger patient cohort.

## Electronic supplementary material

Below is the link to the electronic supplementary material.

Supplementary Material 1

## Data Availability

The data presented in this study are available on specific request from the corresponding author. The data are not publicly available for reasons of data protection and data economy.
