# Supplementary material for: LDH and hemoglobin outperform systemic inflammatory indices as prognostic factors in patients with soft tissue sarcoma undergoing neoadjuvant treatment
Source: BMC Cancer. 2025 Mar 18;25:496. doi: 10.1186/s12885-025-13889-4 (PMC11916319; doi:10.1186/s12885-025-13889-4)
Supplement: Supplementary file 1 — Supplementary Material 1 [file 12885_2025_13889_MOESM1_ESM.docx]

**Supplementary file**

Table 1: Median values of analyzed laboratory parameters

| **Factor** | **Median (Range)** |
| --- | --- |
| Hemoglobin (g/dl) | 13.0 (8.5-17.1) |
| Leukocytes (G/l) | 7.6 (3.8-15.2) |
| Lymphocytes (G/l) | 1.5 (0.6-3.1) |
| Neutrophils (G/l) | 5.3 (2.0-10.9) |
| Monocytes (G/l) | 0.6 (0.1-1.8) |
| Platelets (G/l) | 296 (119-695) |
| Albumin (g/dl) | 4.1 (2.7-5.3) |
| CRP (mg/dl) | 1 (0.1-17.8) |
| LDH (U/l) | 213 (98-655) |
| NLR | 3.7 (1.2-12.2) |
| LMR | 2.3 (0.7-7.4) |
| CLR | 0.6 (0.05-23.5) |
| PLR | 212.7 (68.6-588.7) |

CRP=C-reactive protein, LDH=Lactate dehydrogenase, NLR=Neutrophil-to-lymphocyte ratio, LMR=Lymphocyte-to-monocyte ratio, CLR=CRP-to-lymphocyte ratio, PLR=Platelet-to-lymphocyte ratio

Table 2: Median values of analyzed blood parameters according to different histological subtypes

| Factor | LMS  Median (Range) | LPS  Median (Range) | SS  Median (Range) | UPS  Median (Range) | p-value* |
| --- | --- | --- | --- | --- | --- |
| Hemoglobin (g/dl) | 13.2 (10.7-17.0) | 12.6 (8.5-15.6) | 14.6 (11.1-15.6) | 12.9 (9.0-17.1) | 0.016 |
| Leukocytes (G/l) | 7.3 (3.8-14.1) | 7.5 (4.4-14.7) | 6.5 (4.4-12.1) | 8.9 (4.3-15.2) | 0.018 |
| Lymphocytes (G/l) | 1.5 (0.7-3.1) | 1.4 (0.7-3.1) | 1.4 (1.0-2.8) | 1.5 (0.6-2.3) | 0.97 |
| Neutrophils (G/l) | 4.9 (2.0-10.6) | 5.5 (2.4-10.9) | 4.2 (2.4-7.3) | 6.2 (3.4-10.9) | 0.0013 |
| Monocytes (G/l) | 0.6 (0.3-1.3) | 0.6 (0.2-1.8) | 0.5 (0.4-0.9) | 0.7 (0.1-1.5) | 0.054 |
| Platelets (G/l) | 275 (119-422) | 308 (167-695) | 264 (184-355) | 341 (187-613) | <0.001 |
| Albumin (g/dl) | 4.2 (3.6-4.9) | 4.1 (3.4-5) | 4.5 (3.8-5.3) | 4 (2.7-4.8) | 0.0019 |
| CRP (mg/dl) | 0.74 (0.1-16.7) | 1.1 (0.1-17.8) | 0.35 (0.1-5.1) | 2.1 (0.1-16.7) | 0.0087 |
| LDH (U/l) | 243 (156-655) | 181 (98-318) | 200 (125-273) | 220 (135-432) | <0.001 |
| NLR | 3.13 (1.31-10.38) | 3.79 (1.41-12.25) | 3.38 (1.19-4.44) | 4.57 (1.62-10.13) | 0.029 |
| LMR | 2.82 (0.74-7.39) | 1.99 (1.14-6.16) | 2.68 (1.66-4.64) | 2.08 (1.0-5.94) | 0.11 |
| CLR | 0.58 (0.048-23.52) | 1.06 (0.049-22.82) | 0.152 (0.48-4.25) | 1.36 (0.060-19.19) | 0.031 |
| PLR | 174 (108.21-588.73) | 240 (72.61-442.31) | 188 (68.59-330.10) | 241 (84.35-522.58) | 0.042 |

CRP=C-reactive protein, LDH=Lactate dehydrogenase, NLR=Neutrophil-to-lymphocyte ratio, LMR=Lymphocyte-to-monocyte ratio, CLR=CRP-to-lymphocyte ratio, PLR=Platelet-to-lymphocyte ratio

*Kruskal-Wallis test

Table 3: Statistically significant results of the Dunn’s post-hoc test following Kruskal-Wallis analysis in Table 2

| Factor | Comparison | z-value | p-value (unadjusted) | p-value (adjusted) |
| --- | --- | --- | --- | --- |
| Hemoglobin (g/dl) | LPS vs. SS | -2.97 | 0.0030 | 0.018 |
| Hemoglobin (g/dl) | SS vs. UPS | 2.84 | 0.0046 | 0.027 |
| Leukocytes (G/l) | SS vs. UPS | -2.97 | 0.0030 | 0.018 |
| Neutrophils (G/l) | LPS vs. SS | 2.83 | 0.0047 | 0.028 |
| Neutrophils (G/l) | SS vs. UPS | -3.75 | <0.001 | 0.0011 |
| Platelets (G/l) | LMS vs. UPS | -3.23 | 0.0013 | 0.0075 |
| Platelets (G/l) | SS vs. UPS | -3.34 | <0.001 | 0.0050 |
| Albumin (g/dl) | LPS vs. SS | -2.81 | 0.005 | 0.030 |
| Albumin (g/dl) | SS vs. UPS | 3.71 | <0.001 | 0.0013 |
| CRP (mg/dl) | SS vs. UPS | -3.10 | 0.0020 | 0.012 |
| LDH (U/l) | LMS vs. LPS | 4.64 | <0.001 | <0.001 |
| LDH (U/l) | LPS vs. UPS | -3.00 | 0.0027 | 0.016 |
| CLR | SS vs. UPS | -2.74 | 0.0062 | 0.037 |

CRP=C-reactive protein, LDH=Lactate dehydrogenase, CLR=CRP-to-lymphocyte ratio

Table 4: Univariate analysis of clinicopathological characteristics on event-free and overall survival

|  |  | **EFS** | | **OS** | |
| --- | --- | --- | --- | --- | --- |
| **Factor** | **Strata** | **Sig.** | **Hazard Ratio (95%CI)** | **Sig.** | **Hazard ratio**  **(95%CI)** |
| Age | <62 vs. ≥62 | 0.19 | 0.69 (0.39-1.21) | 0.49 | 0.75 (0.34-1.68) |
| Sex | Female vs. Male | 0.18 | 1.46 (0.84-2.55) | 0.60 | 0.80 (0.35-1.84) |
| Histology | UPS vs. non-UPS | 0.56 | 0.96 (0.86-1.09) | 0.34 | 1.08 (0.92-1.27) |
| Grading | G3 vs. G2 | 0.74 | 0.91 (0.52-1.59) | 0.51 | 1.32 (0.58-3.02) |
| Tumor size (cm) | <10 vs. ≥10 | 0.12 | 0.63 (0.36-1.12) | 0.12 | 0.50 (0.21-1.19) |
| Tumor site | Extremity vs. non-extremity | 0.59 | 0.86 (0.49-1.50) | 0.21 | 1.74 (0.74-4.08) |
| Disease status | Primary vs. Recurrent | 0.030 | 2.59 (1.10-6.08) | 0.88 | 1.12 (0.26-4.79) |
| Resection margins | R0 vs. R1-RX | 0.017 | 0.44 (0.22-0.86) | 0.11 | 0.45 (0.17-1.21) |
| Radiotherapy | Yes vs. No | 0.010 | 0.47 (0.26-0.83) | 0.11 | 0.50 (0.21-1.17) |
| Chemotherapy dose reduction | Yes vs. No | 0.88 | 0.95 (0.48-1.85) | 0.20 | 0.45 (0.13-1.52) |
| Rad. Response (RECIST) | PR/SD vs. PD | 0.10 | 0.57 (0.29-1.12) | **0.0063** | **0.30 (0.13-0.71)** |

UPS=undifferentiated pleomorphic sarcoma, PR=Partial response, SD=Stable disease, PD=Progressive disease

Table 5: Univariate analysis of systemic inflammatory indices on event-free and overall survival

|  |  | **EFS** | | **OS** | |
| --- | --- | --- | --- | --- | --- |
| **Factor** | **Strata** | **Sig.** | **Hazard Ratio (95%CI)** | **Sig.** | **Hazard ratio**  **(95%CI)** |
| Hemoglobin (g/dl) | <13.0 Vs. ≥13.0 | **0.021** | **1.94 (1.10-3.43)** | 0.12 | 1.91 (0.84-4.35) |
| Leukocytes (G/l) | <7.6 vs. ≥7.6 | 0.73 | 1.10 (0.63-1.92) | 0.71 | 0.86 (0.38-1.93) |
| Lymphocytes (G/l) | <1.5 vs. ≥1.5 | 0.54 | 0.82 (0.44-1.54) | 0.37 | 1.50 (0.62-3.62) |
| Neutrophils (G/l) | <5.3 vs. ≥5.3 | 0.66 | 0.87 (0.48-1.60) | 0.86 | 0.92 (0.39-2.19) |
| Monocytes (G/l) | <0.6 vs. ≥0.6 | 0.93 | 0.97 (0.52-1.81) | 0.65 | 0.82 (0.34-1.94) |
| Platelets (G/l) | <296 vs. ≥296 | 0.77 | 1.09 (0.62-1.89) | 0.59 | 1.25 (0.56-2.81) |
| Albumin (g/dl) | <4.1 vs. ≥4.1 | 0.65 | 1.15 (0.63-2.09) | 0.86 | 1.08 (0.45-2.60) |
| CRP (mg/dl) | <1.0 vs. ≥1.0 | 0.69 | 1.12 (0.64-1.95) | 0.78 | 0.89 (0.40-2.0) |
| LDH (U/l) | <213 vs. ≥213 | 0.012 | 0.47 (0.26-0.85) | 0.041 | 0.38 (0.15-0.96) |
| NLR | <3.7 vs. ≥3.7 | 0.99 | 1.00 (0.54-1.87) | 0.10 | 0.45 (0.17-1.17) |
| LMR | <2.3 vs. ≥2.3 | 0.50 | 0.81 (0.43-1.50) | 0.39 | 1.48 (0.61-3.57) |
| CLR | <0.6 vs. ≥0.6 | 0.56 | 1.20 (0.65-2.23) | 0.80 | 0.90 (0.38-2.13) |
| PLR | <212.7 vs. ≥212.7 | 0.46 | 1.26 (0.68-2.35) | 0.34 | 0.65 (0.26-1.57) |
| PAR | <74.6 vs. ≥74.6 | 0.81 | 0.93 (0.51-1.70) | 0.84 | 0.91 (0.37-2.23) |

NLR=Neutrophil-to-lymphocyte ratio, LMR=Lymphocyte-to-monocyte ratio, CLR=CRP-to-lymphocyte ratio, PLR=Platelet-to-lymphocyte ratio, PAR=Platelet-to-albumin ratio, CRP=C-reactive protein, LDH=Lactate dehydrogenase
